# High uric acid or low uric acid which is better for hemodialysis patients?

**DOI:** 10.1080/0886022X.2020.1784227

**Published:** 2020-07-28

**Authors:** Wenyun Wang, Wei Liu, Zhifang Zhao, Yu Zhao, Chengji Zhao

**Affiliations:** a Department of Pediatric Surgery, Second Hospital of Lanzhou University, Lanzhou, PR China; b Department of General Surgery, Hospital of Integrated Traditional Chinese and Western Medicine, Tianshui City, China; c Department of Medicine, Northwest Minzu University, Lanzhou, PR China

I have read with great interest the paper entitled ‘Low serum uric acid levels increase the risk of all-cause death and cardiovascular death in hemodialysis patients’ by Ming et al. [1]. Uric acid (UA) plays an important role in the elimination of nitrogenous compounds and antioxidant in healthy people. The article included 210 hemodialysis (HD) patients with a mean UA of 532.5 ± 137.4 μmol/L, and showed that with a decrease in serum UA(<420 μmol/L), all-cause mortality (log rank χ^2^ = 15.61, *p* = .000), and cardiovascular (CV) mortality (log rank χ^2^ = 14.28, *p* = .000) increased. Each 100 μmol/L increase in serum UA was associated with lower all-cause mortality with a hazard ratio (HR) of 0.792 (0.645-0.972) and lower CV mortality with an HR of 0.683(0.505-0.924) after adjusting for age, sex, and complications. Compared to the lowest quartile (<420 μmol/L), all-cause mortality [HR 0.351(0.132-0.934), *p* = .036] and CV mortality [HR 0.112(0.014-0.925), *p* = .042] were lower in the highest quartile (>644 μmol/L). I pay special attention to the results of this research because it caused me some confusion.

Firstly, a retrospective cohort study by Muela et al. [2]. showed that high UA (≥428 μmol/L) was not associated with either composite CV events or all-cause mortality in 1020 hemodialysis (HD) patients. In the subgroups of patients with diabetes or increased C-reactive protein an elevated UA also did not alter the incidence of events or death. A prospective cohort study of 108 HD patients with a mean UA of 458.2 ± 89.3 μmol/L showed that lower UA (defined as UA level below median) was not associated with all-cause mortality (HR = 1.2, *p* = .2) [3].

Secondly, although the conclusion of the article is consistent with some previous studies, the data in Table 1 still confuses me. According to the KDIGO guidelines [4], the urea nitrogen clearance rate, hemoglobin and serum albumin did not reach the target value in Q1 group for HD patients. All of the indicators are closely related to CV events and all-cause mortality. Therefore, I think the title and conclusion of this article are somewhat misleading.

Finally, Tatsunori et al. [5]. conducted a prospective observational study with 1073 HD patients from 27dialysis centers and followed up for 5 years to reveal UA difference (UAD), which may be the most appropriate reference for controlling UA levels, correlated with all-cause mortality in HD patients. I suppose UAD is the best indicator to evaluate the UA levels.

## References

[1] Li M, Ye Z-C, Li C-M, et al. Low serum uric acid levels increase the risk of all-cause death and cardiovascular death in hemodialysis patients. Ren Fail. 2020;42(1):315–322. 32223483 10.1080/0886022X.2020.1745234 PMC7170277

[2] Muela HCS, De Lima JJG, Gowdak LHW, et al. Prognostic value of serum uric acid in patients on the waiting list before and after renal transplantation. Int J Nephrol. 2015;2015:1–6. 10.1155/2015/375606 PMC4320892 25685556

[3] Ishii T, Ohtake T, Okamoto K, et al. Serum biological antioxidant potential predicts the prognosis of hemodialysis patients. Nephron Clin Pract. 2011;117(3):c230–c236. 20805696 10.1159/000320201

[4] Chan CT, Blankestijn PJ, Dember LM, et al. Dialysis initiation, modality choice, access, and prescription: conclusions from a Kidney Disease: Improving Global Outcomes (KDIGO) Controversies Conference. Kidney Int. 2019;96(1):37–47. 30987837 10.1016/j.kint.2019.01.017

[5] Toida T, Sato Y, Komatsu H, et al. Pre- and postdialysis uric acid difference and risk of long-term all-cause and cardiovascular mortalities in Japanese hemodialysis patients; Miyazaki dialysis cohort study. Blood Purif. 2019;47(suppl 2):50–55. 30943478 10.1159/000496638

